# ^60^Co γ-ray Irradiation Crosslinking of Chitosan/Graphene Oxide Composite Film: Swelling, Thermal Stability, Mechanical, and Antibacterial Properties

**DOI:** 10.3390/polym10030294

**Published:** 2018-03-09

**Authors:** Dawei Zhang, Shuai Yang, Yuanqing Chen, Siyu Liu, Hongtao Zhao, Jiyou Gu

**Affiliations:** 1 College of Materials Science and Engineering, Northeast Forestry University, Harbin 150040, China; ys930509@163.com (S.Y.); cyq0626@126.com (Y.C.); liusiyu7288@163.com (S.L.); 2 Technical Physics Institute of Heilongjiang Academy of Science, Harbin 150086, China; zhaohongtao1976@163.com

**Keywords:** γ-ray irradiation, chitosan, graphene oxide, swelling, thermal stability, mechanical property, antibacterial property

## Abstract

In this paper, chitosan (CS)/graphene oxide (GO) composite films were prepared, and the effect of γ-ray irradiation on the properties of composite films was investigated. The irradiation crosslinking reaction occurred in composite films with the existence of acetic acid, and the properties changed upon the various irradiation dose. The swelling degree of the composite film with 0 wt % GO decreased with the increasing of the irradiation dose, but the swelling degree of which with GO increased instead. The thermal stability increased with the increasing of the irradiation dose, but the effect of the irradiation on the thermal stability weakened as the increasing of the content of GO, due to the enhanced irradiation resistance performance. The tensile strength increased firstly and decreased subsequently with the increasing of the irradiation dose and the content of GO. Composite films showed the enhanced antibacterial activity against *Bacillus subtilis*, compared to *Escherichia coli* and *Staphylococcus aureus*. The antibacterial activity weakened with the increasing of the content of GO. The antibacterial activity was relatively stronger when the irradiation dose was 20 KGy. In addition, the structural, crystal, and morphological properties of composite films were characterized by FT-IR, XRD, and SEM. It is worth noting that the GO was pre-functionalized via KH560 for the better compatibility with CS matrix.

## 1. Introduction

Antibacterial active packaging, as an excellent packaging method, has gained wide attention [1]. Antibacterial packaging means that an antibacterial substance is added into the packaging material or an antibacterial substance is used directly as the packaging material [2]. This type of packaging is of great importance to increase the shelf life of foods of animal origin [3]. As an alternative to antibacterial packaging materials, antibacterial and biodegradable films have been used in several applications due to their capabilities to prevent moisture loss, aromas loss, solute transport, and water absorption in the food matrix or oxygen penetration [4,5].

Natural materials with antibacterial activities such as polysaccharides, proteins, and lipids have been proven to be promising for food packaging applications because of their environmentally friendly and biodegradable properties [6]. Chitosan (CS), derived from chitin, has been proven to be promising for food packaging applications [7,8]. CS is composed of N-acetyl-D-glucosamine and D-glucosamine unit [9]. It contains two kinds of functional groups such as hydroxyl and amino groups that endow various properties to composite materials such as biocompatibility, biodegradability, and antibacterial activity [10]. It is worth noting that CS possesses an excellent film-forming ability, and it can easily be casted into films after dissolution under slightly acidic aqueous conditions. CS has been widely used as a natural source for preparing films in many fields, including packaging, filtration, and some medical applications [11]. The inter- and intra-molecular hydrogen bonding of CS film could improve its mechanical strength [12]. Nevertheless, the water resistance and radiation resistance performances need to be modified.

There have been many researches for the integration of nanofillers into CS based active packaging films, in order to improve the water resistance performance, irradiation resistance performance and mechanical properties [13,14]. GO possesses amounts of oxygen-containing functional groups (such as epoxy, hydroxyl, carbonyl, and carboxyl functional groups [15]) on basal plates and edges, which could be cross-linked by polymers containing amino groups [16] for improving the water resistance performance and mechanical properties of composite films [17]. Meanwhile, the excellent radical scavenging efficiency of GO would improve the irradiation resistance performance of composite films [18]. In addition, before being added into the CS matrix, GO should be functionalized to improve its compatibility with CS.

γ-ray irradiation assures enough high energy for a powerful penetration in the materials [19], and it has been proven to act as an uncomplicated and efficient sterilization method for food package fields [20]. CS is susceptible to degradation when subjected to γ-ray irradiation [21], which could be concerned with the excitation and formation of radical [22]. The irradiation crosslinking of CS-based films has been demonstrated to be an effective treatment for a further enhancement of strength [23]. In addition, γ-ray irradiation is an effective nonthermal physical technology to improve antibacterial activity of CS films [24], and it has also been used in depolymerization of CS in the solid state [25].

In this work, CS/GO composite films were prepared successfully, and the effect of γ-ray irradiation with various doses (0–40 KGy) on the properties of composite films was investigated. The swelling degree of composite films was measured via a swelling test. The chemical structure, crystallinity and morphology were measured using attenuated total refraction Fourier transform infrared spectroscopy (ATR-FT-IR), X-ray diffraction (XRD), and scanning electron microscopy (SEM). Thermogravimetric analysis (TGA) was used to characterize the thermal stability and the mechanical properties were determined via a tensile strength tester. In addition, the antibacterial activity of composite films against *Bacillus subtilis*, *Escherichia coli*, and *Staphylococcus aureus* was investigated.

## 2. Experimental 

### 2.1. Materials

CS (90–91%, deacetylation degree) was obtained from Zhejiang Golden-Shell Pharmaceutical Co., Ltd. (Zhejiang, China), Acetic acid was purchased from Tianjin Zhiyuan Reagent Co., Ltd. (Tianjin, China), GO was prepared in laboratory. 3-Glycidyloxypropyltrimethoxysilane (KH560) was obtained from Aladdin Industrial, Inc. Acetone and ethanol absolute were commercially available from Tianjin Zhiyuan Reagent Co., Ltd. (Tianjin, China), *Bacillus subtilis (B. subtilis*), *Escherichia coli (E. coli*), and *Staphylococcus aureus (S. aureus*) were obtained from the Guangdong Culture Collection Center (Guangdong, China). All of the materials were used as received without further treatment.

### 2.2. Preparation of Functionalized GO

The GO sheets were treated with KH560, which was adopted to compatibility between GO and CS matrix, avoiding GO flocculation. The main procedure of preparation of functionalized GO included three steps: the preparation of graphite oxide, the functionalization of graphite oxide through the addition of KH560, and the dispersion of functionalized graphite oxide. The home-made graphite oxide [26] was washed with acetone to remove the residual water molecule and ions such as Mn^2+^, and then it was centrifuged. Afterward, ethanol with 1 wt % water (100 g) was used as the dispersant for sediment (5 g) and KH560 (0.5 g) was put into the solution. The mixture was heated up to 70 °C with stirring, and the reaction was conducted for 30 min. Then, the mixture was treated with centrifugation. The supernatant was removed, and the sediment was dispersed in appropriate amount of distilled water. Finally, functionalized GO dispersion (0.5 wt %) was obtained with the ultrasonic treatment. There was not deposition in the functionalized GO dispersion for over a month.

### 2.3. Preparation of CS/GO Composite Films

CS powder was dissolved into distilled water with acetic acid (1% *w*/*w*) and stirring to obtain CS solution (1 wt %). Appropriate amount of GO dispersion was added into the CS solution and the mixed solution was stirred to obtain a homogeneous solution. The content of GO was 0 wt %, 1 wt %, 3 wt % and 5 wt % of CS, respectively. The mixed solution was dried at 50 °C for 72 h in a vacuum oven (−0.1 MPa). During the film-forming process, the mixed solution was stirred appropriately, avoiding the formation of bubble.

### 2.4. γ-ray Irradiation

The samples were closed hermetically in brown glass vials, filling with nitrogen, and were irradiated at the ^60^Co γ-ray irradiation source which was supplied by Technical Physics Institute of Heilongjiang Academy of Science, with various γ-ray irradiation doses (0 KGy, 10 KGy, 20 KGy, and 40 KGy).

### 2.5. Swelling Test

Swelling tests were performed for CS/GO composite films using distilled water. The weight of samples before immersing was measured. After gently blotting the film surface with filler paper to remove the absorbed water, the weight of the swollen films was weighed immediately at different time intervals (5, 15, 45, and 90 min). The water absorption of the films was calculated by the following formula:

(1) $$DS(\%)=\frac{W_{s}-W_{0}}{W_{0}}\times 100\%$$

where *DS*(%) was the degree of swelling, *W*_0_ was the weight of films before immersing, and *W_s_* was the weight of swollen films.

### 2.6. Fourier Transform Infrared (FT-IR) Spectroscopy

The chemical structures of CS/GO composite films were investigated by attenuated total refraction Fourier transform infrared spectroscopy (Tensor II FT-IR, Bruker, Billerica, MA, USA). The spectral range was 4000–700 cm^−1^ with a resolution of 4 cm^−1^.

### 2.7. X-ray Diffraction (XRD) Analysis

XRD analysis was used to determine the crystal structures of CS/GO composite films through a XRD analyzer (D/max-2200VPC, Rigaku, Tokyo, Japan). The scanning rate was 5° min^−1^ over a 2θ range of 5°–40°.

### 2.8. Scanning Electron Microscopy (SEM)

The morphology of CS/GO composite films was observed by field emission scanning electron microscopy (Quanta 200, Fei, Hillsboro, OR, USA). The samples were sputter-coated with gold for better conductivity during imaging. The fresh fracture surface was obtained by freezing composite films in liquid nitrogen and then cracking.

### 2.9. Thermogravimetric Analysis (TGA)

The thermal stability of CS/GO composite films was investigated using TGA (TG 209F1 Libra, Netzsch, Selb, Germany) under nitrogen atmosphere. The test temperature was at the range of 30 °C–700 °C and heating rate was 10 °C min^−1^.

### 2.10. Mechanical Properties

Mechanical properties of CS/GO composite films were measured via a tensile strength tester (DSA 502A, Sans, Minneapolis, MN, USA). The composite films were cut into 50 mm × 5 mm (length × width) rectangles, and the sample gauge length was 30 mm. The thickness of each strip was measured. A tensile test was performed at a loading speed of 2 mm min^−1^ at room temperature and 45% relative humidity. Five samples were used to characterize each film.

### 2.11. Antibacterial Property

Antibacterial activity of CS/GO composites films was investigated against *B. subtilis* (ATCC 6633), *E. coli* (ATCC 8739), and *S. aureus* (CMCC 26003) by agar disk diffusion test. Agar plates, filling with Luria-Bertani agar medium, were inoculated with microbial culture. The samples were cut into small rectangle pieces (10 mm × 10 mm) and put on the inoculated agar plates. These plates were then incubated at 37 °C overnight in an incubator. The diameters of zone of inhibition formed were measured via Digimizer software. All the tests were carried out in duplicate.

## 3. Results and Discussion

### 3.1. Swelling Degree

**Figure 1:**
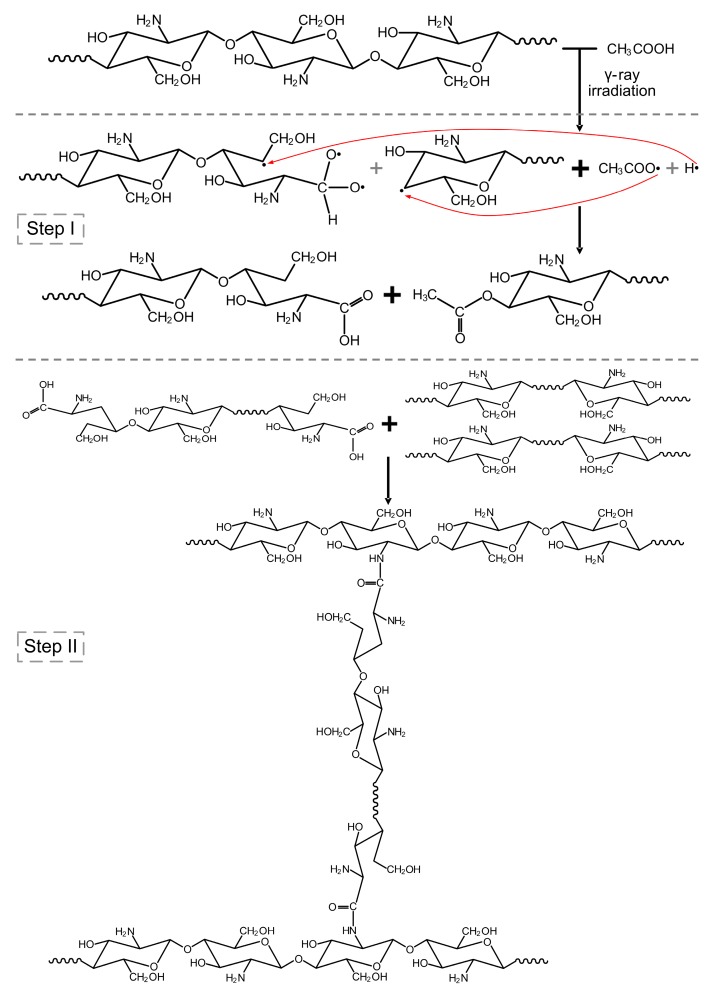
Schematic illustration of the potential mechanism of irradiation crosslinking reaction of composite films with the existence of acetic acid.

The CS/GO composite films were immersed into the acetic acid (1% *w/w*) aqueous solution. When the content of GO was 0%, the film before irradiating was dissolved. After irradiating, the film just swollen, but did not dissolve. Meanwhile, once the GO was added into the CS matrix, the films was swollen with or without irradiation. The groups on GO sheets reacted with the groups on CS molecules [27], promoting to the formation of crosslinking structure. Besides, with the existence of acetic acid, γ-ray irradiation could also induce to the occurrence of crosslinking reaction. The potential mechanism of irradiation crosslinking reaction is shown in Figure 1.

**Figure 2:**
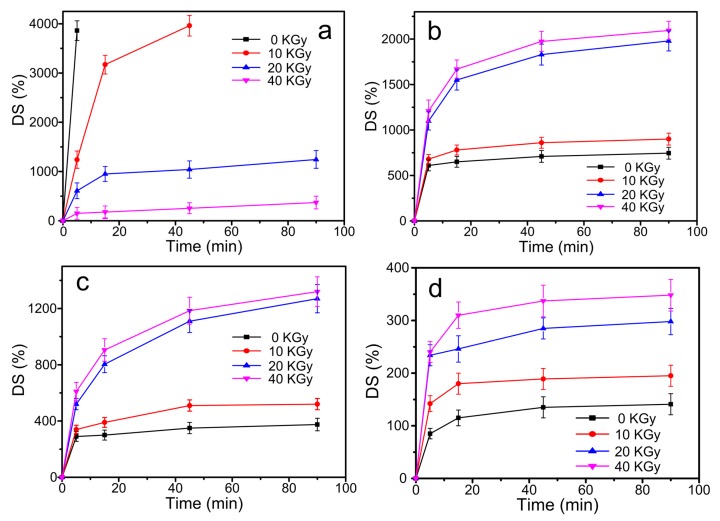
Swelling curves of chitosan (CS)/graphene oxide (GO) composite films with different content of GO: (**a**) 0 wt %; (**b**) 1 wt %; (**c**) 3 wt %; (**d**) 5 wt % under the various irradiation doses.

In order to reveal the effect of γ-ray irradiation on the crosslinking degree of CS/GO composite films, the swelling test was carried out, and swelling curves of CS/GO composite films with various irradiation doses (0, 10, 20, and 40 KGy) are shown in Figure 2. Figure 2a–d showed swelling curves of CS/GO composite films with different content of GO (0 wt %, 1 wt %, 3 wt %, and 5 wt %, respectively). In Figure 2a, it was clear that as the increasing of irradiation dose, the swelling degree of composite film with 0 wt % GO decreased significantly. The swelling degree was up to 4000% in 5 min without irradiating. Nevertheless, when the irradiation dose was up to 40 KGy, the swelling degree decreased to 300%. There was no obvious increment when the immersed time continued to increase. This could be attributed to the occurrence of crosslinking reaction inducing by irradiating with the existence of acetic acid. The formation of reticular structure of the CS chains limited the entering of the water molecules. As a result, the swelling degree decreased. In Figure 2b–d, the swelling degree of composite films was far less than it of composite film with 0 wt %, which could be attributed to that the dispersion of GO in CS matrix acted as an effective crosslinking point. The increased crosslinking degree decreased the swelling degree. It was worth noting that when composite films were irradiated, the swelling degree increased. It was due to that the absorption of radical by GO sheets hindered the occurrence of crosslinking reaction, but the rupture of CS chains still continued. As a result, the swelling degree increased.

### 3.2. FT-IR

**Figure 3:**
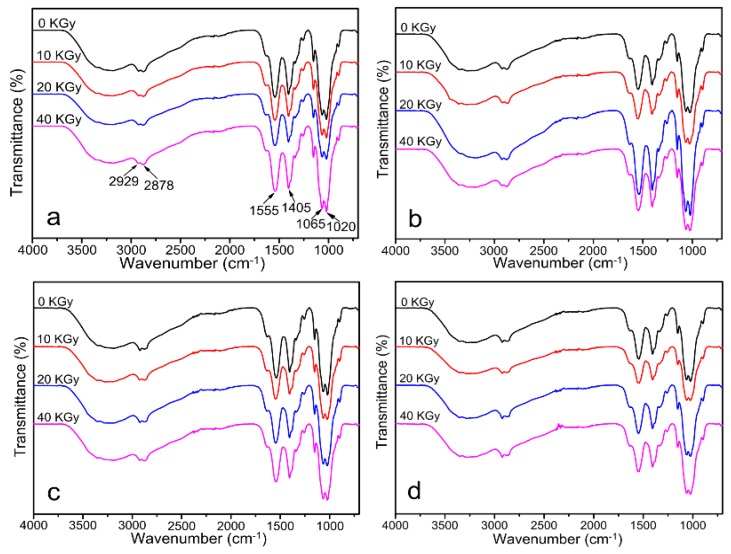
Fourier Transform Infrared (FT-IR) spectra of CS/GO composite films with different content of GO: (**a**) 0 wt %; (**b**) 1 wt %; (**c**) 3 wt %; (**d**) 5 wt % under the various irradiation doses.

FT-IR spectra of CS/GO composite films with various irradiation doses (0, 10, 20, and 40 KGy) are shown in Figure 3. Figure 3a–d showed FT-IR spectra of CS/GO composite films with different content of GO (0 wt %, 1 wt %, 3 wt %, and 5 wt %, respectively). In Figure 3a, the peaks at 1065 cm^−1^ and 1020 cm^−1^ could be concerned with the C–OH stretching vibration in the CS molecule. The peak at 1405 cm^−1^ was due to the vibrational bending of C–H bond in methylene group. The peak at 1555 cm^−1^ corresponded to the bending frequency of the amide bond of CS [28]. The peak at 2878 cm^−1^ was assigned to the stretching vibration of C–H [29]. After the irradiation, the saccharide ring was opened, and it reacted with the irradiated acetic acid. The peak of the newly formed ester and amide groups would be covered by the groups existed in system before. As a result, there were not changes of peak position as the increasing of irradiation dose. Meanwhile, there were not obvious changes among Figure 3a–d. It could be attributed to that the epoxy and carboxyl groups on GO sheets acted with the amino groups on CS chains. The peaks of the secondary amino groups and amide groups would be covered by the peaks of amino groups and amide groups that existed in system before [26].

### 3.3. XRD

**Figure 4:**
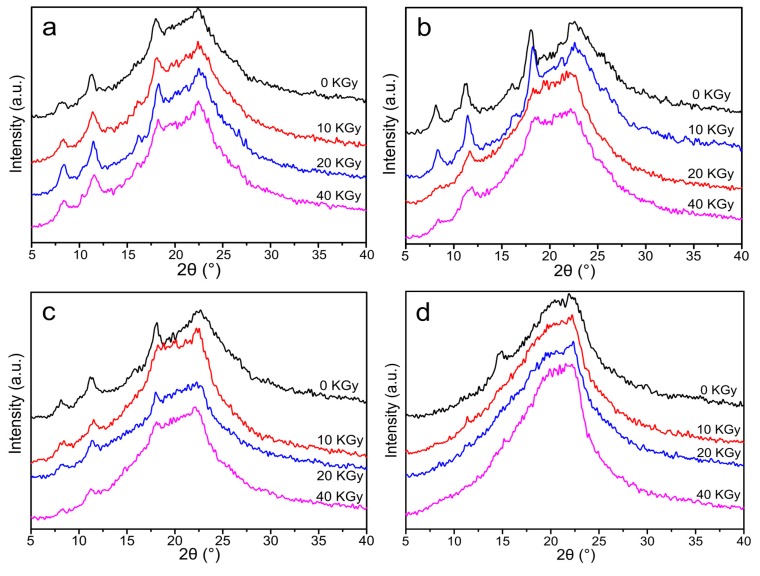
X-ray diffraction (XRD) patterns of CS/GO composite films with different content of GO: (**a**) 0 wt %; (**b**) 1 wt %; (**c**) 3 wt %; (**d**) 5 wt % under the various irradiation doses.

XRD patterns of the CS/GO composite films with various irradiation doses (0, 10, 20 and 40 KGy) are shown in Figure 4. Figure 4a–d showed XRD patterns of CS/GO composite films with different content of GO (0 wt %, 1 wt %, 3 wt % and 5 wt %, respectively). In Figure 4a, it could be obviously observed that the main peaks were at 2θ = 8.3°, 11.4°, 18.2° and 22.6°. The crystalline of CS included Crystalline I and Crystalline II [30]. The peak of Crystalline I was at 2θ = 11.4° and the peak of Crystalline II was at 2θ = 22.6°. As the increasing of irradiation dose, the peak at 2θ = 11.4° became broad due to the occurrence of crosslinking reaction. Nevertheless, the peak position did not shift obviously which could be obtained that the crosslinking reaction merely occurred at amorphous region. In Figure 4b, when the irradiation dose was 20 KGy, the peaks at 2θ = 8.3° and 2θ = 22.6° disappeared and peaks at 2θ = 11.4° and 2θ = 18.2° decreased significantly. In Figure 4c, when the irradiation dose was up to 20 KGy, the peak at 2θ = 11.4° decreased drastically and another peaks almost disappeared. Meanwhile, in Figure 4d, once the irradiation dose was up to 10 KGy, the only peak at 2θ = 14.8° disappeared. It could indicate that the addition of GO destroyed the crystalline structure of CS via the occurrence of crosslinking reaction between groups on CS chains and GO sheets. Besides, the increased irradiation dose decreased the crystallinity to some extent.

### 3.4. Morphology of Composite Films

**Figure 5:**
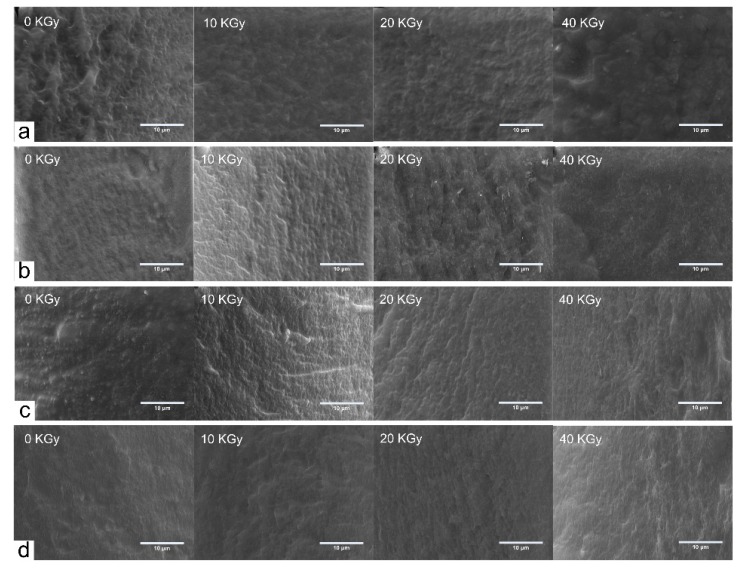
Scanning electron microscopy (SEM) images of CS/GO composite films with different content of GO: (**a**) 0 wt %; (**b**) 1 wt %; (**c**) 3 wt %; (**d**) 5 wt % under the various irradiation doses.

Figure 5 shows SEM images of CS/GO composite films with various irradiation doses (0, 10, 20 and 40 KGy). Figure 5a–d showed SEM images of CS/GO composite films with different content of GO (0 wt %, 1 wt %, 3 wt %, and 5 wt %, respectively). In Figure 5a, it could be observed that comparing with the original smooth fracture surface, there were some slight warping on the surface after irradiating. From Figure 5b–d, there appeared some wrinkle with the addition of GO, which could be attributed to the slight assemble of GO. With the treatment of γ-ray irradiation, the fracture surface became smooth and the wrinkle disappeared. This indicated that the internal structure of composite films changed, induced by the irradiation. The original protruded GO sheets would be stripped down due to the removal of groups, resulting in the smooth fracture surface.

### 3.5. TGA

**Figure 6:**
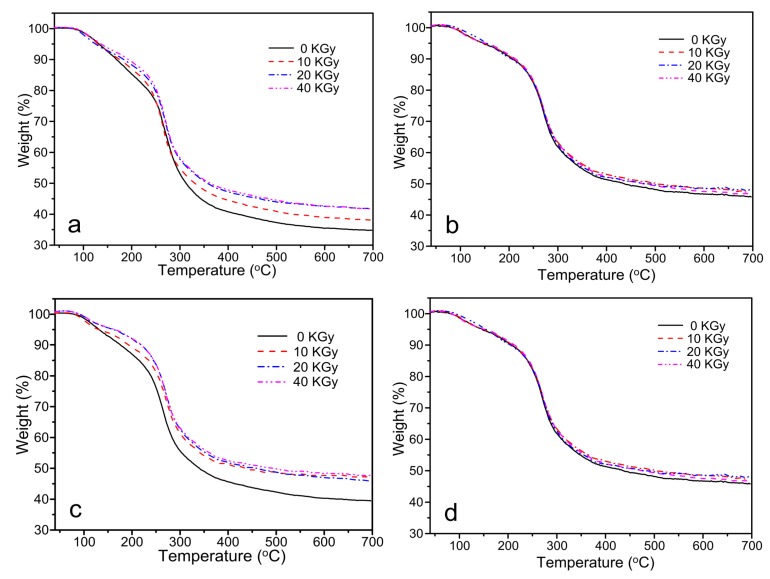
Thermogravimetric analysis (TGA) analysis of CS/GO composite films with different content of GO: (**a**) 0 wt %; (**b**) 1 wt %; (**c**) 3 wt %; (**d**) 5 wt % under the various irradiation doses.

TGA curves of the CS/GO composite films with various irradiation doses (0, 10, 20 and 40 KGy are shown in Figure 6. Figure 6a–d showed TGA curves of CS/GO composite films with different content of GO (0 wt %, 1 wt %, 3 wt %, and 5 wt %, respectively). In Figure 6a, it was clear that a serious weight loss (Stage I) appeared at the range of 90 °C–120 °C, which could be concerned with the loss of bound water absorbed in CS molecule. Another significant degradation (Stage II) occurred at about 220 °C–270 °C, which was concerned with the degradation of CS chains. As the increasing of irradiation dose, the initial weight at Stage II increased, and the residual weight at 700 °C increased, which could be attributed to that the crosslinking reaction occurred between the radical inducing by irradiating. The chain structure was transformed into reticular structure and as result, the residual increased. In Figure 6b,c, as the addition of GO, the crosslinking reaction between groups on CS molecule and GO sheets occurred. After irradiating, the crosslinking and degradation reaction inducing by irradiating occurred simultaneously. When the irradiation dose was 10 KGy, the crosslinking was the dominance and as a result, the thermal stability increased. When continued to increase the irradiation dose (20 KGy and 40 KGy), the degradation of CS chains became the major process and the thermal stability decreased. The same tendency was reflected on the swelling curves. When the content of GO increased to 5 wt %, the absorption of radical inducing by GO acted as a determined element to hinder the occurrence of crosslinking reaction. The effect of irradiation became really small, and as a result, the difference of irradiation dose (10, 20 , and 40 KGy) would not affect significantly the thermal stability of composite films. This could indicate that the enhanced radiation resistance performance of composite films was obtained through the addition of a high content (5 wt %) of GO.

### 3.6. Mechanical Properties

**Figure 7:**
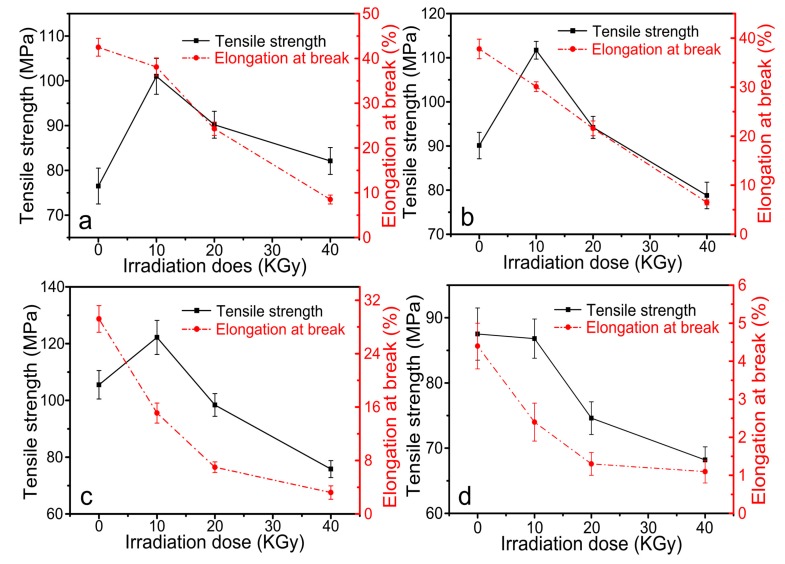
Tensile strength and elongation at break of CS/GO composite films with different content of GO: (**a**) 0 wt %; (**b**) 1 wt %; (**c**) 3 wt %; (**d**) 5 wt % under the various irradiation doses.

Tensile test results of CS/GO composite films with various irradiation doses (0, 10, 20 and 40 KGy) are shown in Figure 7. Figure 7a–d showed tensile strength and elongation at break of CS/GO composite films with different content of GO (0 wt %, 1 wt %, 3 wt %, and 5 wt %, respectively). In Figure 7a, the tensile strength of composite film with 0 wt % GO increased firstly, decreased subsequently and the maximum occurred at the irradiation dose was 10 KGy. After irradiating, the crosslinking reaction between the radical inducing by irradiating occurred and the tensile strength increased. Nevertheless, with the increasing of irradiation dose, the increased crosslinking degree resulted in the inhomogeneous dispersion of crosslinking point. As a result, the effect of stress concentration occurred, which accelerated the destruct of internal structure during the stretching process. Thus, the tensile strength decreased. Elongation at the break of composite film with 0 wt % decreased significantly with the increasing of irradiation dose, which could be the result of the effect of the crosslinking reaction. The increased crosslinking points decreased the distance between the contiguous crosslinking points and as a result, elongation at break decreased. Meanwhile, the rupture of CS chains would also contribute to the process to some extent.

In Figure 7b,c, as the addition of GO, the tensile strength of composite films increased firstly and decreased subsequently and the elongation at break decreased drastically. The radical inducing by irradiating was absorbed by GO sheets and the dominance of decreased elongation at break changed from the crosslinking reaction to the rupture of CS chains. When the content of GO was up to 5 wt %, the absorption of radical by GO became a determined element. The crosslinking reaction became very small, but the degradation reaction continued. As a result, the tensile strength decreased with the increasing of irradiation dose. In addition, as the increasing of content of GO, the tensile strength of composite films increased first, decreased subsequently, and elongation at break decreased constantly.

### 3.7. Antibacterial Activity Assay

**Figure 8:**
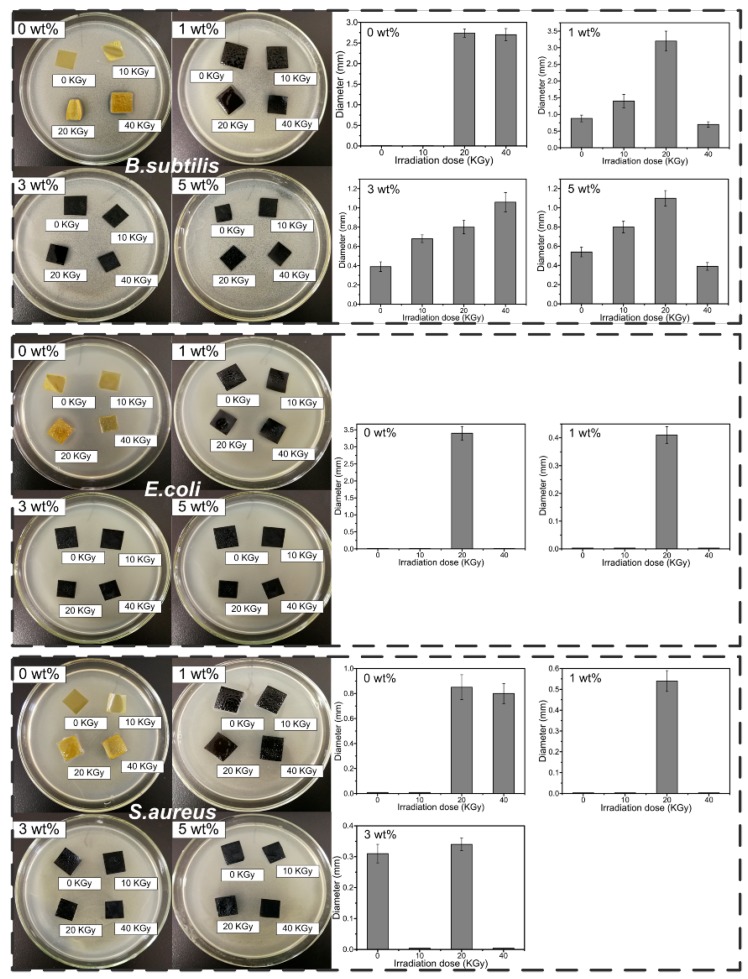
Inhibition zone measurement results (Photographic images and Diameter statistics of inhibition zone) of CS/GO composite films with different content of GO (0 wt %, 1 wt %, 3 wt %, and 5 wt %) under the various irradiation doses against *B. subtilis*, *E. coli*, and *S. aureus*.

The antibacterial activity of CS/GO composite films with various irradiation doses (0, 10, 20 and 40 KGy) against *B. subtilis*, *E. coli* and *S. aureus* was investigated by inhibition zone measurements, and the results are shown in Figure 8. It could be obtained clear that the antibacterial activity of composite films against *B. subtilis* was stronger than *E.coli* and *S.aureus*. For *B. subtilis*, when the irradiation dose was up to 20 KGy, composite films shown the stronger relatively antibacterial activity. The diameter of inhibition zone was 2.74 and 2.70 mm, corresponding to the irradiation dose of 20 and 40 KGy, when the content of GO was 0 wt %. When the content of GO was 1 wt %, the diameter was 3.48 mm, corresponding to the irradiation dose of 20 KGy. It could be attributed that as the increasing of irradiation dose, the rupture of CS chains and the radical inducing by irradiating increased the amount of positive charge on CS chains, resulting in the enhanced antibacterial activity. As the increasing of content of GO, the antibacterial activity of composite films weakened. The addition of GO would decrease the amount of positive charge on CS chains due to the crosslinking reaction between groups on GO sheets and on CS molecule. As a result, the antibacterial activity of composite films weakened. Meanwhile, as the increasing of content of GO, the absorption of radical by GO might contribute to the phenomenon to some degree. For *E. coli*, the diameter of inhibition zone was 3.48 mm and 0.4 mm, corresponding to the content of GO was 0 wt % and 1 wt %, when the irradiation dose was 20 KGy. There was not the appearance of inhibition zone at other conditions. As for *S. aureus*, when the irradiation was 20 KGy, composite films showed the stronger relatively antibacterial activity.

## 4. Conclusions

In this paper, CS/GO composite films were prepared, and the effect of γ-ray irradiation with various irradiation doses on the properties of composite films was investigated. The crosslinking reaction induced by irradiating occurred with the existence of acetic acid. In the swelling test, when the content of GO was 0%, the swelling degree decreased significantly with the increasing irradiation dose. Nevertheless, with the addition of GO, the increased irradiation dose increased the swelling degree. There were not obvious changes in FTIR spectra, which indicated that the peak position did not change with the addition of GO and the treatment of γ-ray irradiation. XRD curves showed the significant decreased crystallinity with the increase of GO content and the increase of irradiation dose. SEM images showed that the occurrence of wrinkle as the addition of GO. However, the fracture surface became smooth and the wrinkle disappeared after irradiating. TGA indicated that as the increasing of GO, the thermal stability increased. Increasing the irradiation dose also increased the thermal stability due to the occurrence of crosslinking reaction. It was worth noting that when the content of GO increased to 5 wt %, the effect of irradiation became very small, and as a result, the difference of irradiation dose would not significantly affect the thermal stability. This could indicate that the enhanced radiation resistance performance of composite films was obtained through the addition of high content (5 wt %) of GO. In the tensile tests, the tensile strength increased first and subsequently decreased with the increasing of content of GO and irradiation dose. The maximum occurred at the content of GO of 3 wt % and the irradiation dose of 10 KGy. Meanwhile, the elongation at the break decreased constantly with the increasing of content of GO and the irradiation dose. In the antibacterial activity test, composite films showed the enhanced antibacterial activity against *B. subtilis*, *E. coli*, and *S. aureus*. When the irradiation dose was 20 KGy, the antibacterial activity was relatively stronger. As the GO content increased, the antibacterial activity weakened. In conclusion, CS/GO composite films with the treatment of γ-ray irradiation proved to be a promising approach to be used widely in antibacterial active packaging.
